# Estimation of Eyelid Pressure Using a Blepharo-Tensiometer in Patients with Functional Nasolacrimal Duct Obstruction

**DOI:** 10.1155/2018/8792102

**Published:** 2018-08-08

**Authors:** Jinsoo Kim, Sang-Mok Lee, Youn Joo Choi, Min Joung Lee

**Affiliations:** ^1^Department of Ophthalmology, Hallym University College of Medicine, Hallym University Sacred Heart Hospital, Anyang, Republic of Korea; ^2^Department of Cornea, External Disease & Refractive Surgery, HanGil Eye Hospital, Catholic Kwandong University College of Medicine, Incheon, Republic of Korea; ^3^Department of Ophthalmology, Hallym University College of Medicine, Hallym University Kangdong Sacred Heart Hospital, Seoul, Republic of Korea

## Abstract

**Purpose:**

To compare the eyelid pressure between patients with functional nasolacrimal duct obstruction (FNLDO) and normal controls using blepharo-tensiometer, and to evaluate the relationship between eyelid pressure and the outcomes of silicone intubation (SI) in patients with FNLDO.

**Study design:**

Prospective case-control study.

**Methods:**

We enrolled 36 eyes of 36 patients with suspected FNLDO who underwent SI and 36 healthy eyes of age-matched controls. One eye of each patient with FNLDO was randomly selected for analysis. The eyelid pressure was estimated using a blepharo-tensiometer and compared between the control and FNLDO groups. The relationship between eyelid pressure and clinical variables was analyzed. The outcomes of SI were assessed at 6 months after surgery using subjective and objective criteria.

**Results:**

The eyelid pressure was significantly lower in the FNLDO group than in the control group (*P*=0.008). In the control group, the eyelid pressure was correlated with age (*P*<0.001) and lower eyelid laxity (*P*=0.016). In the FNLDO group, the eyelid pressure was only correlated with age (*P*<0.001). The success rate of SI for FNLDO was 69.4% (25 of 36 eyes). The eyelid pressure was higher in the surgical success subgroup than in the failure subgroup, although the difference was not statistically significant (*P*=0.08).

**Conclusions:**

Our results suggest that the eyelid pressure measured using a blepharo-tensiometer has a diagnostic value since it is decreased in patients with FNLDO. The role of eyelid pressure as a possible predictor of the outcomes of SI for FNLDO should be investigated in further studies. This trial is registered with KCT0002828.

## 1. Introduction

Functional nasolacrimal duct obstruction (FNLDO) can be defined as the presence of epiphora with a patent lacrimal drainage system according to an irrigation test and without any evidence of potential causative factors, including ocular surface abnormalities, lid margin abnormalities, eyelid laxity, and punctal or canalicular stenosis, although several different definitions have been published [1–6]. It is well recognized that the action of the orbicularis muscle and eyelid dynamics are important contributing factors for FNLDO because movements of the orbicularis oculi muscle create hydrostatic pressure and facilitate tear flow [7]. However, a quantitative method for measuring the function of the orbicularis muscle has not been established.

Recently, a commercially developed device with tactile pressure sensors, known as a blepharo-tensiometer, was introduced to evaluate the tension exerted by the eyelids [8]. The blepharo-tensiometer has been used to investigate the mechanical friction between the eyelids and the ocular surface, particularly in patients with dry eye and lid-wiper epitheliopathy [9, 10]. The sensor measures the compression pressure between the eyelid and conjunctiva during eyelid closure. Accordingly, the eyelid pressure directly reflects the power of the orbicularis oculi muscle.

In the present study, we evaluated the clinical usefulness of the eyelid pressure measured using a blepharo-tensiometer for the diagnosis and management of FNLDO by comparing measurements between patients with FNLDO and normal controls. We also assessed the relationship of eyelid pressure with clinical variables and determined whether this parameter is a useful predictor of the outcomes of silicone intubation (SI) performed for FNLDO.

## 2. Patients and Methods

This prospective case series was approved by the Institutional Review Board of Hallym University Sacred Heart Hospital (No. 2016-I151). The protocols of the study adhered to the tenets of the Declaration of Helsinki, and written informed consent was obtained from all subjects.

### 2.1. Study Subjects

We enrolled patients with suspected FNLDO who underwent SI at the oculoplasty clinic in Hallym University Sacred Hospital between January 2017 and December 2017. We also enrolled age-matched controls. All participants in this study were Koreans who underwent comprehensive ophthalmological examinations and assessments of the lacrimal drainage system, including tear meniscus height measurements, the fluorescein dye disappearance test (FDDT), the lacrimal syringing test, lacrimal probing, nasal endoscopic examinations, and dacryocystography (DCG). Dacryoscintigraphy (DSG) was not routinely performed.

The tear meniscus height was measured using a slit lamp beam. A narrow, straight, vertical beam with a height of 0.2 mm or 1.0 mm at 16x magnification was applied after instillation of one drop of fluorescein. We classified the tear meniscus height as “high” when the superior edge of the meniscus was equal to or more than half the height of the 1.0 mm beam (≥0.5 mm). If the meniscus height was less than half the height of the 1.0 mm beam, the 0.2 mm beam was applied. The meniscus was defined as “normal” when its height was less than the height of the 0.2 mm beam. The meniscus was classified as “moderate” when its height was ≥0.2 mm and <0.5 mm [11]. The FDDT findings were interpreted based on the residual amount of fluorescein dye at 5 minutes after instillation and were scored from 0 (complete clearance) to 4+ (no or little clearance). A score of 2+ to 4+ was considered to indicate positivity. Lid laxity was evaluated by the anterior distraction test, which measures the distance from the globe to the lower eyelid margin when the lower lid is pulled away from the globe. Body mass index (BMI) was defined as the body mass (kg) divided by the square of the body height (m).

The criteria for the diagnosis of FNLDO were as follows: (1) bilateral epiphora with a high tear meniscus, (2) positive FDDT findings, (3) adequate passage without reflux or resistance in the lacrimal irrigation test, and (4) absence of focal stenosis or narrowing of the lacrimal drainage system on DCG. One eye from each participant was randomly selected for inclusion in this study. The age-matched controls were recruited from patients who had visited our clinic for unilateral epiphora and were diagnosed with unilateral partial NLDO or total NLDO. The contralateral eye with no tearing symptoms and no evidence of anatomical obstruction in FDDT, the lacrimal syringing test, and DCG, served as a control.

The exclusion criteria were as follows: evidence of reflex tearing due to ocular surface abnormalities; previous history of ocular or orbital trauma, surgery, chemotherapy, or radioiodine treatment; facial nerve palsy; lower eyelid or punctal malpositioning; and severe horizontal eyelid laxity (anterior distraction over 10 mm). Reflex tearing was considered when any corneal epithelial lesion, including punctate epithelial erosion, was observed on slit lamp examination or when the tear breakup time was less than 5 seconds.

### 2.2. Eyelid Pressure Measurement Using a Blepharo-Tensiometer

The eyelid pressure was measured for all subjects using a commercially developed measurement system with tactile pressure sensors (DigiTacts Single Point Sensors; Pressure Profile Systems, Inc, Los Angeles, California, USA) [10]. The lower eyelid pressure was measured by a single ophthalmologist for all subjects. Following the application of 0.5% proparacaine hydrochloride ophthalmic solution for topical anesthesia (Alcaine®, Alcon, Fort Worth, TX, USA), a disposable contact lens (−0.5 diopters, PureVision, Bausch & Lomb, Rochester, NY, USA) was placed on the cornea. The pressure sensor was covered with a disposable polyethylene protective cap (20 *µ*m thickness), and a zero point was adjusted. The pressure sensor was inserted between the soft contact lens and the conjunctival side of the lower eyelid. The subjects were asked to close their eyes for over 10 s, and the eyelid pressure was measured at the plateau phase.

### 2.3. Silicone Intubation Procedure and Evaluation of Surgical Outcomes

SI was performed under local anesthesia by a single surgeon (MJL). The inferior meatus was packed with 4% lidocaine with 1 : 1,000 epinephrine, and the medial part of the lower and upper eyelids was infiltrated with 2% lidocaine with 1 : 100,000 epinephrine. Following punctum dilation, probing was performed with a Bowman probe (Probe Lachrymal BOWMAN 04-05, Inami, Tokyo, Japan) to confirm the absence of resistance in the lacrimal sac and the nasolacrimal duct (NLD). If there was any difficulty in passing the probe, the case was excluded from the study. A silicone Crawford tube was passed from the upper and lower puncta to the inferior meatus through the canaliculi, lacrimal sac, and NLD. The olive tip on the end of the tube was retrieved with a hook. The stents were tied together with 6-0 black silk and fixed to the lateral wall of the nasal cavity with minimal traction using 5-0 polypropylene. The patients were followed up at 1 week and 1, 3, and 6 months after SI. Subsequent examinations were also performed by the same surgeon (MJL). The surgical outcomes were assessed subjectively and objectively at 6 months. Subjective success was defined as the absence of tearing, assessed using Munk's score [12]; 0: no epiphora, 1: occasional epiphora requiring drying or dabbing less than twice a day, 2: epiphora requiring dabbing two to four times per day, 3: epiphora requiring dabbing five to ten times per day, 4: epiphora requiring dabbing more than ten times daily or constant tearing. The presence of grade 0 or grade 1 epiphora was also a criterion for success. Objective success was defined as a decrease in the tear meniscus height. Surgical success was achieved when both the subjective and objective success criteria were satisfied.

### 2.4. Statistical Analysis

All statistical analyses were performed using SPSS Statistics 22.0 (IBM, Armonk, NY, USA) and GraphPad Software (GraphPad Prism®, Inc., La Jolla, CA, USA). With regard to the comparison of demographic and clinical characteristics, Mann–Whitney *U* tests were used for continuous variables, and Fisher's exact tests were used for categorical variables. The relationship between the eyelid pressure and clinical variables was analyzed using Pearson's correlation analysis. Continuous values are presented as means ± standard deviations. A *P* value of <0.05 was considered statistically significant.

## 3. Results

### 3.1. Subject Characteristics

The present study included 36 eyes of 36 patients with FNLDO and 36 healthy eyes of 36 age-matched controls. The demographic and clinical characteristics of patients in the FNLDO and control groups are shown in Table 1. The mean lower eyelid laxity was 5.25 ± 1.11 and 5.67 ± 1.45 mm for the control and FNLDO groups, respectively; the difference was not significant (*P*=0.245). The proportion of patients with an eyelid laxity of ≥7 mm was 13.9% in the control group and 27.8% in the FNLDO group, but the difference was not significant (*P*=0.245). However, the lower eyelid pressure measured using the blepharo-tensiometer was significantly lower in the FNLDO group (14.0 ± 0.4 mmHg) compared to the control group (16.1 ± 0.6 mmHg; *P*=0.008). There was no significant difference in the BMI between the control and FNLDO groups (*P*=0.094).

### 3.2. Correlation between the Lower Eyelid Pressure and Clinical Variables

When the eyelid pressure was examined according to age, a significant decrease was observed with an increase in age in both the control (*r* = −0.73, 95% confidence interval (CI): −0.86 to −0.52, *P*<0.001) and FNLDO groups (*r* = −0.63, 95% CI: −0.79 to −0.37, *P*<0.001) (Table 2). The BMI was not significantly correlated with the eyelid pressure in either group. The lower eyelid pressure was inversely correlated with lower eyelid laxity in the control group (*r* = −0.40, *P*=0.016), but not in the FNLDO group (*r* = −0.27, *P*=0.107).

### 3.3. Surgical Outcomes of SI for FNLDO

In total, 25 (69.4%) patients achieved surgical success and 11 (30.6%) experienced failure. There were no major complications. The follow-up period was significantly longer for the surgical success group (Table 3). Age, sex, laterality, BMI, and lower eyelid laxity showed no differences between the two groups. However, the lower eyelid pressure was higher in the surgical success subgroup (14.6 ± 2.26 mmHg) than in the surgical failure subgroup (12.8 ± 2.95 mmHg), although this difference was not statistically significant (*P*=0.08, Mann–Whitney *U* test).

## 4. Discussion

In the present study, we found that the eyelid pressure measured using a blepharo-tensiometer was significantly lower in eyes with FNLDO than that in healthy eyes. Other demographic and clinical variables that could possibly predict FNLDO including eyelid laxity were also analyzed, and they did not show any significant difference between the affected and control eyes. In particular, we focused on the horizontal lower eyelid laxity and compared the two groups with regard to the mean value and proportion of patients with an eyelid laxity of ≥7 mm; there was no significant difference. Our results suggest that the eyelid pressure represents the contraction power of the orbicularis muscle and reflects the lacrimal pump function more sensitively than horizontal lower eyelid laxity. However, our findings were inconsistent with those of a previous Greek study where it was reported that the lacrimal clearance examined using DSG differed according to the horizontal eyelid laxity [1]. Both studies used the eyelid distraction test which measures the amount of posteroanterior retraction of the lower eyelid from the corneal surface caused by manual pinching to evaluate the horizontal eyelid laxity. The eyelid distraction test is a subjective test with high interobserver reliability, although it is widely used by oculoplastic clinicians. In addition, we excluded patients with excessive eyelid laxity (anterior distraction over 10 mm) from our study because of the possibility of eyelid and punctal malpositioning.

Tear flow is believed to be determined by the balance between the hydrostatic pressure of tears and the resistance of the lacrimal drainage system. In eyes with FNLDO, the resistance of the lacrimal drainage system is negligible, so the tear flow is mainly determined by the hydrostatic pressure, which depends on gravity, the capillary phenomenon, and the lacrimal pump. The mechanism of the lacrimal pump remains a debatable topic although several theories have been proposed. However, it is indisputable that contraction of the orbicularis muscle is an important contributing factor for tear drainage [13, 14]. Several modalities are used to establish a differential diagnosis of FNLDO in patients with epiphora, such as lacrimal scintigraphy, magnetic resonance imaging (MRI), and ultrasound biomicroscopy (UBM) [1, 15–18]. Conventionally, lacrimal scintigraphy is used to investigate FNLDO. A diluted tracer is dropped into the lower conjunctival fornix and serial images are acquired as it moves down the lacrimal system [19]. This technique can show the physiological flow of tears and has been considered as a standard method for FNLDO diagnosis. However, it has some limitations for use in clinical practice including the requirement of special equipment, potential radiation hazards, and low resolution of images. Dynamic MRI with or without radioisotopes has also been used to investigate the lacrimal pump by some researchers [16, 18]. It is a minimally invasive method, but it is not commonly available, is expensive to use in the clinic, and provides early results. Recently, the lacrimal pump function was tested using UBM in patients with facial nerve palsy, and the results revealed that fluid turbulence was absent or decreased according to the degree of facial nerve palsy [15]. UBM is simple and easy to perform in the clinic and provides high-resolution images, although it can show only qualitative results. Compared with all these modalities, the blepharo-tensiometer is a minimally invasive, office-based method with no radiation hazards, and it provides quantitative results. However, it cannot show anatomical details and cannot aid in the investigation of theories concerning lacrimal pump function.

In the present study, we also evaluated the correlations of the eyelid pressure with age, BMI, and lower eyelid laxity. The eyelid pressure decreased significantly with increasing age in both the control and FNLDO groups, consistent with previous findings [8, 10]. With regard to the horizontal laxity, an inverse correlation with the eyelid pressure was observed only in the control group, not in the FNLDO group. This can be explained by the fact that the eyelid pressure is generally low in patients with FNLDO. However, it remains unclear whether FNLDO is caused by a low eyelid pressure. In addition, we investigated the correlation between the eyelid pressure and BMI on the basis of a previous report suggesting that an increase in weight is associated with eyelid laxity; however, no significant correlation was found in both groups [20]. Racial differences in the body proportion may have affected the results. The mean BMI of our subjects (23.9 kg/m^2^ in the control group and 22.9 kg/m^2^ in the FNLDO group) was much lower than that (29 kg/m^2^) in the previous study [20].

The treatment options for FNLDO include SI, endonasal or external DCR, and other eyelid procedures for the correction of laxity, although the standard protocol is somewhat controversial. Some researchers reported that external or endonasal dacryocystorhinostomy is an appropriate option for FNLDO with a surgical success rate of up to 90% [21, 22]. However, others have advocated SI as a primary treatment option for FNLDO because it is much less invasive compared with other techniques. Previous studies have reported a surgical success rate of approximately 70% for SI [2, 6]. However, the prognostic factors for surgical success are not well known. In this study, the overall success rate for SI was 69.4%, which is comparable with that reported in previous studies. Furthermore, the eyelid pressure was higher in the surgical success subgroup than in the surgical failure subgroup, although the difference was not statistically significant. Further large-scale studies are desirable to confirm whether the eyelid pressure is a predictor of the outcomes of SI for FNLDO. There are some possible theories that can explain the role of SI in the treatment of FNLDO. Moscato et al. [2] postulated that SI allows mucosal dilation that can lower the resistance of the lacrimal outflow system and increase the lacrimal flow volume. In addition, augmentation of the capillary phenomenon is an important mechanism to facilitate the gravitational flow. Therefore, we leave the stent for over 6 months with the intention of permanent placement if the patient does not want to undergo removal. Six months after surgery, we evaluated the surgical outcomes. For patients with surgical success, we suggested two options: remove or keep the stent. If the patient wanted to maintain the stent placement, we allowed the patient to keep it, and regularly followed up with the patient every 4 to 6 months.

The limitations of our study include the small number of cases and the short follow-up period after SI. In addition, the examiner who measured the eyelid pressure was not blinded to the groups, and the medial and lateral canthal laxity were not measured. Despite these limitations, we believe that the eyelid pressure measured using a blepharo-tensiometer is a useful parameter for the diagnosis and management of FNLDO.

In conclusion, our results suggest that the eyelid pressure measured using a blepharo-tensiometer, which offers a minimally invasive and office-based measurement method, is decreased in patients with FNLDO. Furthermore, a high eyelid pressure could be a predictor of successful SI. Thus, this measure can be used for the diagnosis and management of FNLDO. We believe that our preliminary findings study can serve as the basis for future large-scale, double-blind studies to confirm the clinical usefulness of the blepharo-tensiometer for the diagnosis and management of FNLDO. In addition, the role of the eyelid pressure as a possible predictor of the outcomes of SI for FNLDO should be investigated in further studies.

## Figures and Tables

**Table 1:** Demographic characteristics of patients with functional nasolacrimal duct obstruction and age-matched controls with healthy eyes.

	Normal control (*n*=36)	FNLDO^a^ patient (*n*=36)	*P* value
Age (years)	61.2 ± 8.9	61.4 ± 8.8	0.940
Female, *n* (%)	26 (72.2)	28 (77.8)	0.786
Right side, *n* (%)	20 (55.6)	21 (58.3)	1.000
Body mass index (kg/m^2^)	23.9 ± 2.6	22.9 ± 1.9	0.094
Lower eyelid laxity (mm)	5.25 ± 1.11	5.67 ± 1.45	0.245
Lower eyelid laxity ≥7 mm, *n* (%)	5 (13.9)	10 (27.8)	0.245
Lower eyelid pressure (mmHg)	16.1 ± 0.6	14.0 ± 0.4	0.008

^a^Functional nasolacrimal duct obstruction.

**Table 2:** Correlations of the lower eyelid pressure with age, body mass index, and lower eyelid laxity.

Lower eyelid pressure	Age	Body mass index	Lower eyelid laxity
*Normal control*			
*r* (95% CI)	−0.73 (−0.86 − −0.52)	−0.24 (−0.53 – 0.11)	−0.40 (−0.65 − −0.07)
*P*	<0.001	0.16	0.016

*Patients with* ^a^ *FNLDO*			
*r* (95% CI)	−0.63 (−0.79 − −0.37)	0.05(−0.28 – 0.39)	−0.27(−0.56 – 0.07)
*P*	<0.001	0.74	0.107

**Table 3:** Comparison of clinical factors between surgical success and failure groups after silicone intubation in patients with functional nasolacrimal duct obstruction.

	Surgical success (*n*=25)	Surgical failure (*n*=11)	*P* value
Follow-up (months)	9.56 ± 5.75	6.09 ± 1.70	0.03
Age (years)	61.2 ± 8.0	61.4 ± 11.3	0.946
Female, *n* (%)	21 (84.0)	7 (63.6)	0.214
Right side, *n* (%)	14 (56.0)	7 (63.6)	0.729
Body mass index (kg/m^2^)	22.6 ± 2.05	23.5 ± 1.57	0.218
Lower eyelid laxity (mm)	5.76 ± 1.48	5.45 ± 1.44	0.660
Lower eyelid laxity ≥7 mm, *n* (%)	8 (32.0)	2 (18.2)	0.458
Lower eyelid pressure (mmHg)	14.6 ± 2.26	12.8 ± 2.95	0.08

## Data Availability

The authors can provide the raw data upon request under the permission of the Institutional Review Board of Hallym University Sacred Heart Hospital.
